# Combination of ligand and structure based virtual screening approaches for the discovery of potential PARP1 inhibitors

**DOI:** 10.1371/journal.pone.0272065

**Published:** 2022-09-12

**Authors:** Mohammad M. Al-Sanea, Garri Chilingaryan, Narek Abelyan, Michael Mamikonyan, Hayk Gasparyan, Sargis Hovhannisyan, Abdelrahman Hamdi, Ahmed R. Ali, Samy Selim, Ahmed A. B. Mohamed

**Affiliations:** 1 Department of Pharmaceutical Chemistry, College of Pharmacy, Jouf University, Sakaka, Saudi Arabia; 2 Institute for Molecular Medicine, Huntington Beach, California, United States of America; 3 Institute of Molecular Biology of NAS RA, Yerevan, Armenia; 4 Institute of Biomedicine and Pharmacy, Russian-Armenian University, Yerevan, Armenia; 5 Department of Mathematics and Mechanics, Yerevan State University, Yerevan, Armenia; 6 Department of Pharmaceutical Organic Chemistry, Faculty of Pharmacy, Mansoura University, Mansoura, Egypt; 7 Department of Medicinal Chemistry, Faculty of Pharmacy, Mansoura University, Mansoura, Egypt; 8 Department of Clinical Laboratory Sciences, College of Applied Medical Sciences, Jouf University, Sakaka, Saudi Arabia; Alagappa University, INDIA

## Abstract

Poly (ADP-ribose) polymerase 1 (PARP1) has high therapeutic value as biomolecular target for research and development of small molecules with antineoplastic activity, since it is upregulated in many cancers, especially in ovarian and BRCA 1/2 mutated breast cancers. Decades of investigation of PARP inhibitors (PARPi) have led to the approval of several drug compounds, however clinical application of PARPi in cancer therapy is limited due to a number of factors, including low selectivity, weak affinity and undesired side effects. Thus, identification of novel drug-like chemical compounds with alternatives to the known PARPi chemical scaffolds, binding modes and interaction patterns with amino acid residues in the active site is of high therapeutic importance. In this study we applied a combination of ligand- and structure-based virtual screening approaches with the goal of identification of novel potential PARPi.

## Introduction

Poly (ADP-ribose) polymerase 1 (PARP1) has a crucial role in many biological processes, including differentiation, proliferation, transcription, DNA repair and inflammation. PARP1 enzyme has special therapeutic interest since it is upregulated in many cancers, including ovarian, breast, skin, colorectal, lung and others [1–6]. The activation of PARP1 is caused by the damage in DNA and facilitates the recruitment of a set of proteins to ensure the reparation process of single-strand DNA breaks. There are four functional domains in the PARP1 enzyme: (N)-terminal DNA-binding domain (DBD), auto-modification domain (AD), WGR domain, and the carboxy (C)-terminal catalytic domain (CAT) [7, 8]. CAT has ADP-ribosyl transferase (ART) subdomain, which includes two amino acid residues that are important for NAD^+^ binding (tyrosine and histidine), and also a glutamic acid residue for polymerase activity [8–11]. The main mechanism of action of PARPi involves their interaction with the catalytic domain of PARP enzyme. The interaction of PARPi with the β-NAD+ binding site of PARP1 leads to the inhibition of its enzymatic activity. Dysfunction of the repair mechanism drives the increase of errors and ultimately results in the cell death [12–15]. PARPi are the first approved drugs with an antineoplastic activity, whose mechanism of action targets the DNA damage response in BRCA1/2 mutated breast and ovarian cancers [5, 2, 16]. Around 88 clinical trials of PARP inhibitors listed in clinicaltrials.gov have been completed and several PARPi have been approved for the treatment of BRCA-mutated ovarian, breast and pancreatic cancer. Currently more clinical trials are registered and being conducted on examining the use of PARP inhibitors as an anti-cancer therapy in chemo-resistant germline or somatic BRCA1/2 mutated breast, ovarian, lung, and pancreatic cancers [17]. PARPi have clinically presented promising efficiency against different carcinomas, especially against breast and ovarian cancer [18, 19]. Clinical application of PARPi in cancer therapy is limited due to a number of factors, including low selectivity, weak affinity and undesired side effects [20]. Identification of novel drug-like chemical compounds with alternatives to the known PARPi chemical scaffolds, binding modes and interaction patterns with amino acid residues in the active site is of high therapeutic importance. There are currently several FDA-approved PARP inhibitors available, including olaparib (Lynparza, AstraZeneca), Rucaparib (Rubraca, Clovis Oncology Inc.), Niraparib (ZEJULA, Tesaro Inc.) and talazoparib (TALZENNA, Pfizer Inc.). Olaparib was the first PARP inhibitor that entered clinical trials and been approved by FDA and EMA as a monotherapy for BRCA-mutated for the treatment of advanced, germline BRCA mutated ovarian cancer [21]. Niraparib has been approved for the maintenance treatment of reoccurring ovarian, fallopian and primary peritoneal carcinomas [22]. Talazoparib was approved for the treatment of germline BRCA1/2-mutated advanced or metastatic HER2-negative breast cancer in 2018 [23]. These FDA-approved compounds and other investigational compounds (veliparib [24] and fluzoparib [25]) are nonselective PARP1 inhibitors that also inhibit PARP2 with similar potency. Besides catalytic inhibition of PARP protein, other important mechanism of PARPi involves trapping of PARP1 protein on the damaged site of DNA, causes blockade of replication and transcription fork and ultimately leads to death of cancer cells [26]. Among all aforementioned compounds, talazoparib is widely known as the most potent trapping inhibitor, with 100-fold more efficiency in PARP trapping than olaparib. The ability to trap the PARP1 on DNA differs than the catalytic inhibition and is of clinical importance as inhibitory mechanism leading to the death of cancer cells. Therefore, in our study we applied the ligand-based computational methods for the identification of novel potential drug-like chemical scaffolds, alternatives to the known PARPi (talazoparib, in particular) in combination with structure-based methods for binding pose and affinity prediction and analysis of the results.

## Materials and methods

### Preparation of library of compounds and ligand-based virtual screening

The purchasable dataset of ZINC20 [27] (http://zinc20.docking.org/) was used as a library for virtual screening of potential PARP inhibitors (~13.3 million compounds). Dataset was filtered using the PAINS filter [28]. Thereafter the dataset was filtered based on the shape similarity to the talazoparib using OpenEyes scientific ROCS software [29]. ROCS is a powerful ligand-based virtual screening tool which was used for the rapid identification of potentially active compounds by shape comparison to the talazoparib. ROCS performs shape-based overlays, which are based on a description of the molecules as atom-centered gaussian functions, of a candidate molecule to a reference molecule. Two scores was used to evaluate the tested compounds to the reference: 1) the Shape Tanimoto coefficient is used to rank molecules against the query molecule based on their shape similarity and 2) The Color Tanimoto score, that counts appropriate overlap of groups that describe properties, such as H-bond donors and acceptors, cations, anions, rings, etc.

### Structure-based virtual screening

Virtual screening was performed using the ICM-PRO software package [30]. ICM-PRO demonstrated high accuracy based on the multiple benchmark studies for molecular docking and virtual screening software among both academic and commercial software [31, 32]. The docking algorithm of the ICM-PRO software is based on the Monte Carlo minimization approach [33]. The scoring function of ICM-PRO software is a weighted sum that includes van der Waals energy of the ligand-target interactions, internal force field energy of the ligand, hydrogen bonding interactions, hydrogen bond donor-acceptor desolvation energy, hydrophobic free energy gain and others. The 3D structure of PARP in complex with one of the most potent inhibitors (talazoparib) was downloaded from Protein Data Bank (PDB ID: 7KK3) and used for virtual screening.

### Re-scoring using MMGBSA approach

The algorithm for MMGBSA binding energy calculation includes three stages: 1) parametrization of the receptor and ligand, 2) minimization and 3) MMGBSA calculations [34]. General Amber Force Field (GAFF) [35] with AM1-BCC charge model [36] was used for small molecule parametrization, while ff14SB [37] force field is used to describe protein parameters. The algorithm for the binding energies calculations is based on a freely-available AmberTools suite (www.ambermd.org/AmberTools) and the full code is available at the following link: https://github.com/sahakyanhk/iPBSA.

Clusterization dendrogram and figures of chemical structures were obtained using ICM-PRO. Comparative analysis of physicochemical features of the identified compounds with reference ligand (talazoparib) was performed using OpenEye ROCS’s ROCSReport utility (https://www.eyesopen.com/rocs).

### Molecular dynamics simulations

AMBER20 package was used to carry out molecular dynamics simulations [38]. Protein parametrization was performed using the ff14SB force field, while for ligand parametrization GAFF with AM1-BCC charge model was used. Minimized conformations of complexes of PARP1 protein with selected compounds obtained from previous stage (MMGBSA re-scoring) were used as starting positions for corresponding simulations. The complexes were solvated in TIP3P water model and Na+/Cl− ions at 150 mM concentration [39]. The Monte Carlo barostat [40] with reference pressure at 1 bar and Langevin thermostat [41] with collision frequency (gamma_ln) 2 ps^−1^ were used to keep the temperature at 310.15 K. The Particle Mesh Ewald (PME) method with 1.0 nm cutoff was used for the long-range electrostatic interactions. Each simulation consisted of 5 ns of system minimization and equilibration and 100 ns of conventional molecular dynamics simulation. Finally, for every simulation, binding energies were calculated using MMPBSA.py program [42], using 250 snapshots with equal intervals collected from the last 20 ns of simulation. RMSD was calculated as indicator of stability of studied complexes during simulations. Besides, RMSF analysis was performed to measure the average atomic flexibility of the Cα atoms of the docked complexes.

## Results and discussion

10,374,250 compounds remained as the result of filtration of the initial database (~13,276,808 compounds) using PAINS filter. These 10,374,250 compounds were additionally filtered for shape similarity to the reference ligand (talazoparib) using OpenEye’s ROCS tool and 500,000 compounds with highest shape similarity to the reference compound were selected for the following molecular docking study.

Molecular docking of the selected 500,000 compounds against the binding site of the catalytic domain of PARP1 enzyme (PDB ID: 7KK3, chain C) was performed using ICM-PRO software. As the result of molecular docking, 168 compounds demonstrated higher docking scores in comparison to the reference ligand (S1 Table).

**Fig 1:**
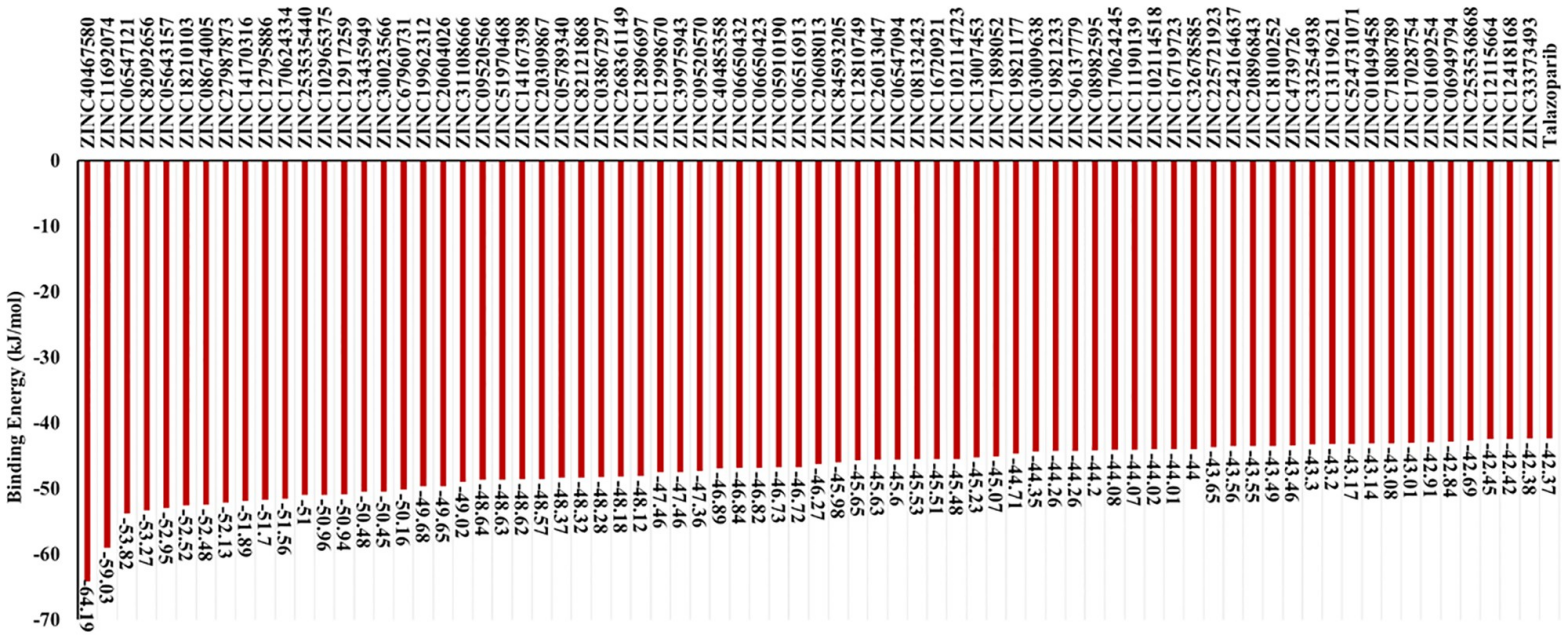
MMGBSA binding energies. Binding energies of identified compounds and talazoparib (reference compounds) as the result of MMGBSA re-scoring.

With the goal of validation of obtained results, binding energies of identified 168 compounds were recalculated using MMGBSA method. MMGBSA re-scoring showed that only 74 compounds demonstrated close or higher binding energies in comparison to talazoparib (Fig 1).

**Fig 2:**
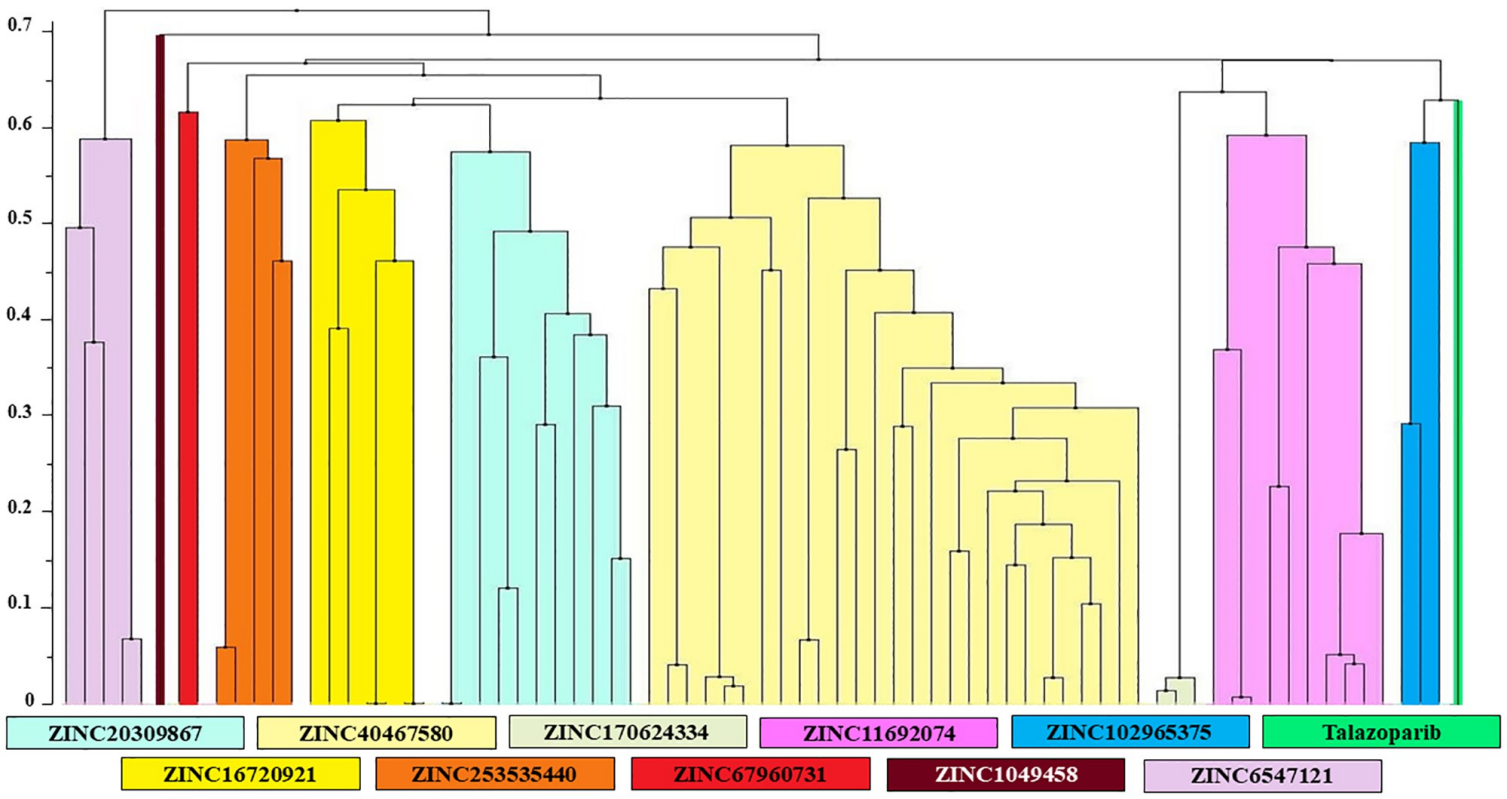
Clusterization dendrogram. Clusterization of the 74 identified compounds with higher binding energies in comparison to talazoparib. For each cluster ZINC ID of the representative compounds with highest estimated binding energy is labeled.

These 74 compounds can be divided into 10 clusters based on their chemical similarity (Fig 2).

The compound with the highest estimated binding energy (ZINC40467580, -64.19 kJ/mol) lies within the biggest cluster (Fig 2). Identified compounds include derivatives of the dihydrophthalazine, dimethoxyphenyl, quinazolin, imidazole, tetrahydroisoquinoline, sulfonamide, fluoroaniline and others.

**Fig 3:**
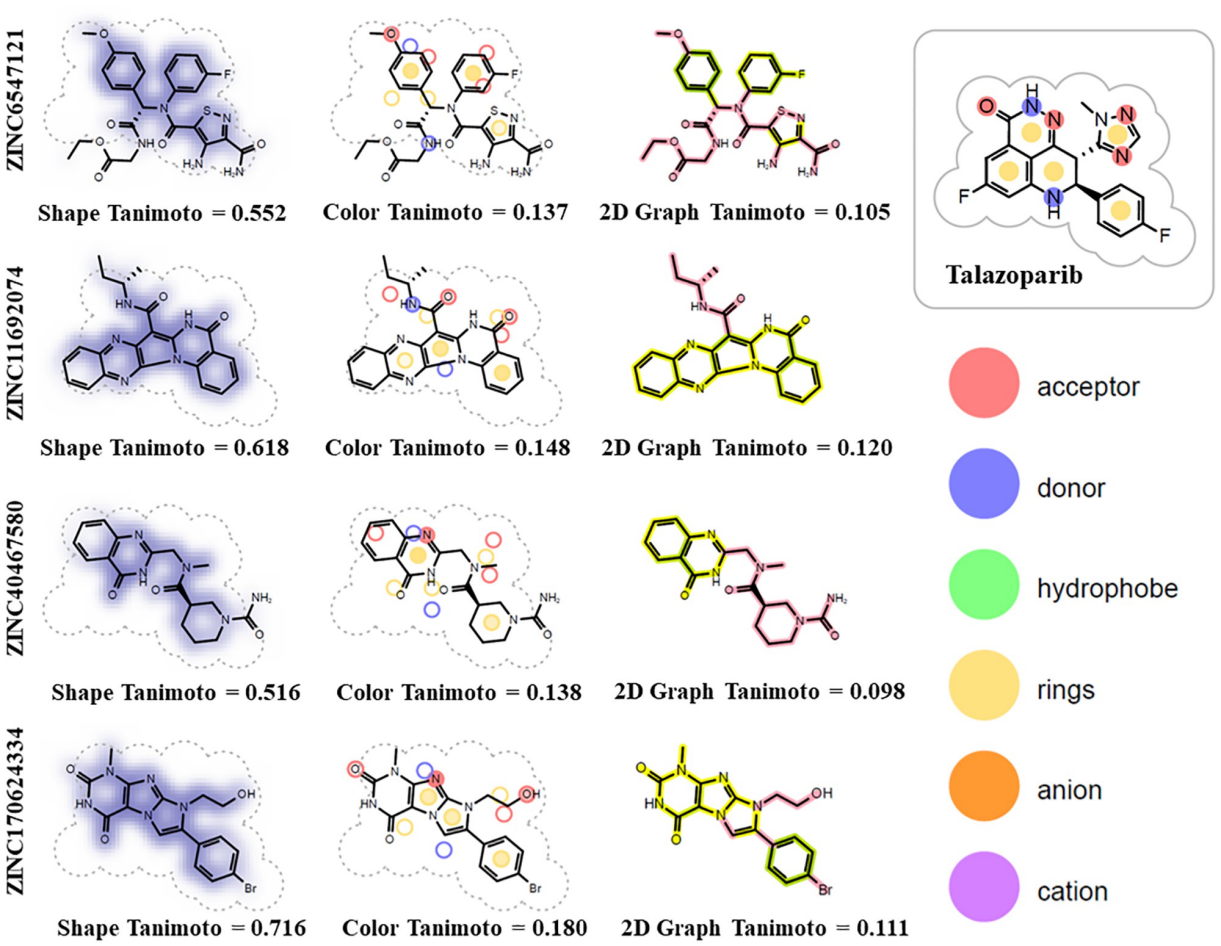
Chemical similarity analysis. Chemical similarity of top 4 identified compounds with talazoparib. In 2D Graph similarity score, pink color highlights parts of the hit molecule that are dissimilar to the talazoparib, while “yellow to dark green” color gradient highlights the bonds similarity.

Similarity analysis of the four selected compounds (representative compounds of clusters with the highest estimated binding energies) and the reference drug-compound (talazoparib) demonstrated significant differences in both chemical structure and functional groups (Fig 3).

ZINC170624334(7-(4-bromophenyl)-6-(2-hydroxyethyl)-4-methylpurino[7,8-a]imidazole-1,3-dione) has the highest shape similarity (0.716) to the talazoparib among four analyzed compounds. ZINC11692074 (N-[(2S)-butan-2-yl]-9-oxo-2,10,14,21-tetrazapentacyclo[11.8.0.02,11.03,8.015,20] henicosa-1(21),3,5,7,11,13,15,17,19-nonaene-12-carboxamide), ZINC40467580 ((3R)-3-N-methyl-3- N-[(4-oxo-3H-quinazolin-2-yl)methyl]piperidine-1,3-dicarboxamide) and ZINC6547121, (ethyl 2- [[(2S)-2-(N-(4-amino-3-carbamoyl-1,2-thiazole-5-carbonyl)-3-fluoroanilino)-2-(4-methoxyphenyl)acetyl]amino]acetate) demonstrated following values of shape similarity to the talazoparib: 0.618, 0.516 and 0.552, respectively.

**Fig 4:**
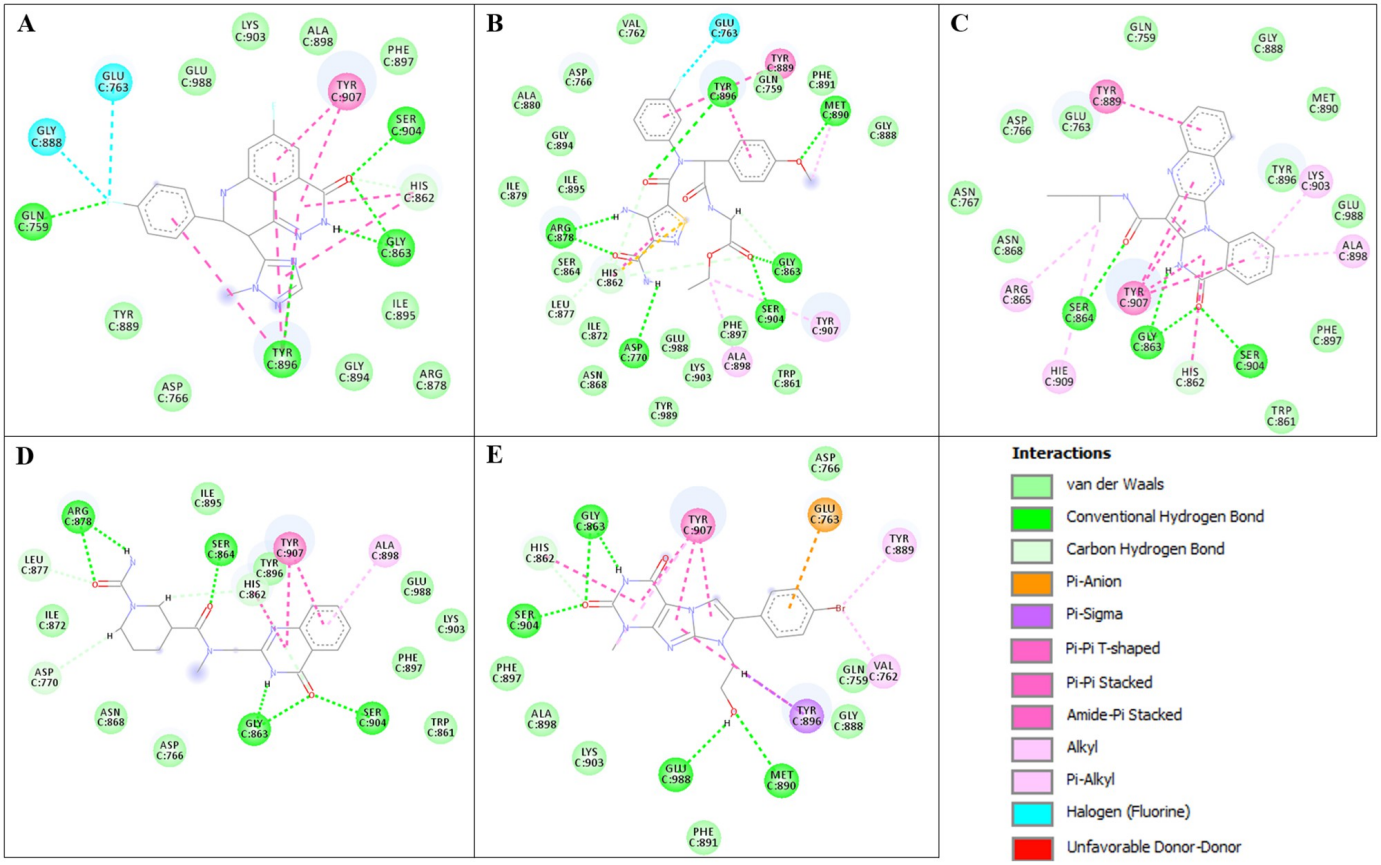
2D interaction diagrams. Interaction of PARP1 active site’s amino acid residues with A) Talazoparib, B) ZINC6547121, C) ZINC11692074, D) ZINC40467580, E) ZINC170624334.

Interaction of the selected compounds and reference ligand with the amino acid residues of the PARP1 active site are presented in Fig 4. Talazoparib forms 5 conventional hydrogen bonds with the following amino acid residues of the PARP1’s active site: GLN 759, GLY 863, TYR 896, SER 904. ZINC6547121 forms 7 conventional hydrogen bonds with 6 amino acid residues, from which 3 are similar to talazoparib (SER 904, GLY 863, TYR 896) and other 3 (ASP 770, ARG 878, MET 890) are different. ZINC11692074 forms 4 conventional hydrogen bonds with 3 amino acid residues of PARP1’s active site: GLY 863, SER 864 and SER 904, where SER 904 and GLY863, again, are common interacting residues for both compounds. ZINC40467580 forms 6 conventional bonds with 5 amino acid residues: GLY 863, SER 864, ARG 878 and SER 904, while ZINC170624334 forms 5 conventional hydrogen bonds with GLY 863, MET 890, SER 904 and GLU 988. Again, only SER 904 and GLY 863 are common interacting amino acid residues of PARP1 involved in hydrogen bonds formation for ZINC40467580, ZINC170624334 and reference drug compound talazoparib. Thus, all five studied compounds, including reference drug compound talazoparib form conventional hydrogen bonds with the SER 904 and GLY 863. Formation of hydrogen bonds with ASP 770 and GLU 988 is unique for the ZINC6547121 and ZINC170624334 respectively. ZINC6547121 and ZINC40467580 both form conventional hydrogen bonds with ARG 878. ZINC6547121 and ZINC170624334 interact with the MET 890.

**Fig 5:**
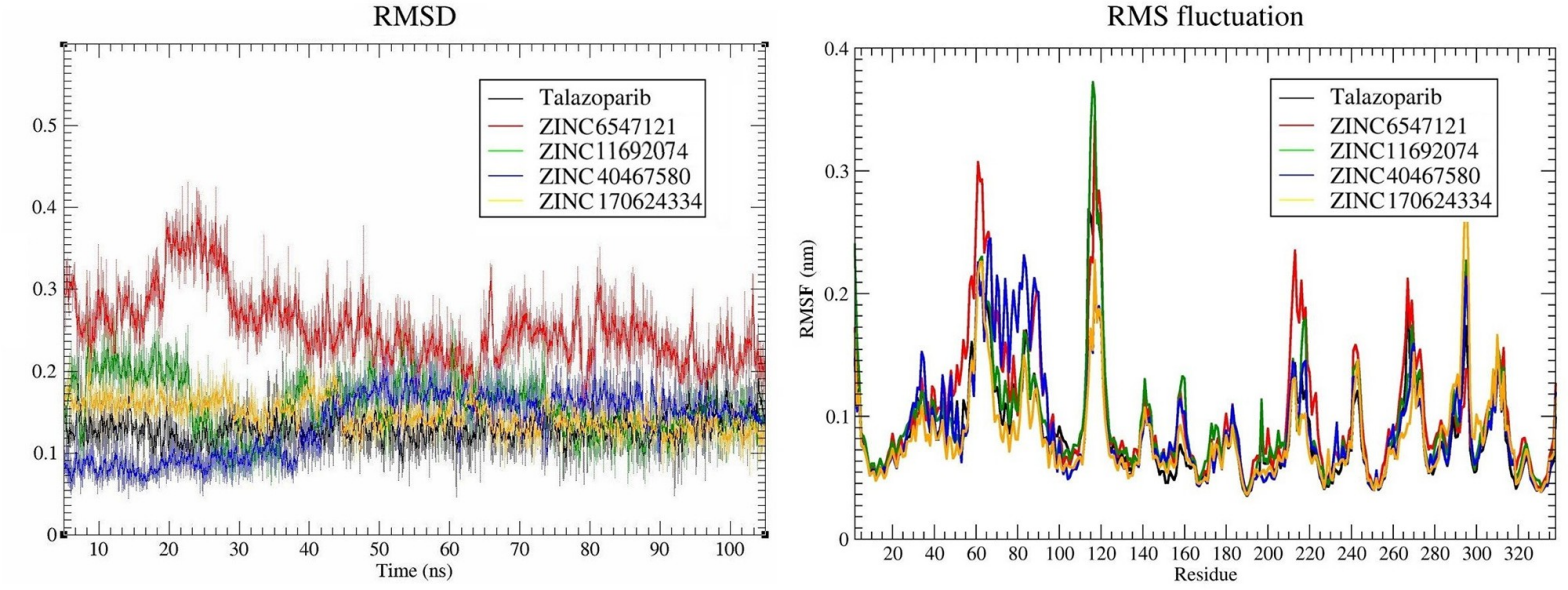
RMSD values of the top 4 compounds and talazoparib; and RMSF values of the PARP1 protein during performed molecular dynamics simulations.

To obtain information on stability of interactions and binding tendency of selected compounds and reference ligand, 5 molecular dynamics simulations were performed. Based on the RMSD values of studied compounds (<0.2 nm) and RMSF values of PARP1 amino acid residues during the performed molecular dynamics simulations, all studied interactions demonstrated stability (Fig 5).

**Fig 6:**
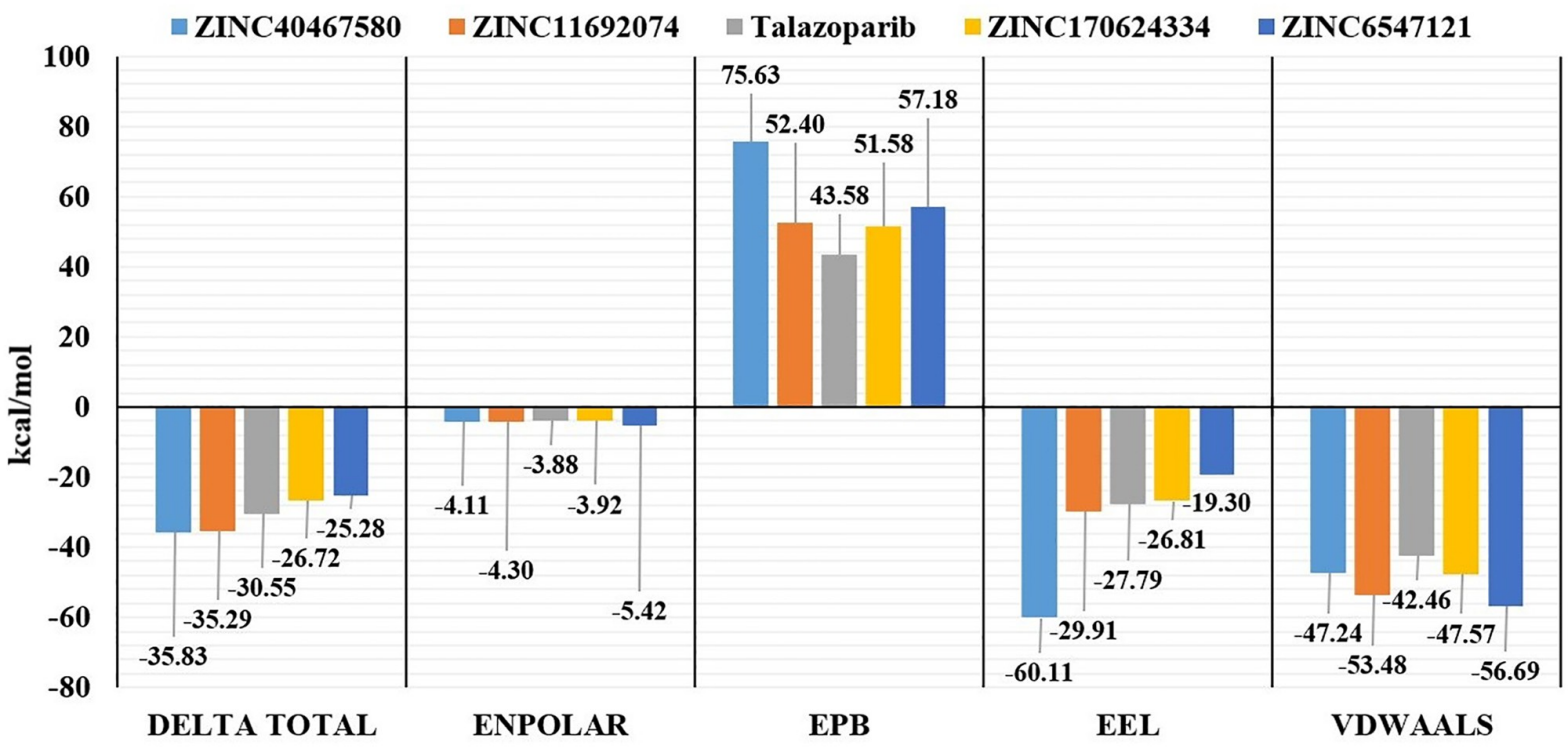
MMPBSA binding energies of studied compounds and talazoparib. Delta total—final estimated binding free energy, Enpolar—nonpolar contribution to the solvation free energy calculated by an empirical model. EPB—the electrostatic contribution to the solvation free energy calculated by PB, VDWAALS—van der Waals contribution, EEL—electrostatic energy.

Binding free energies of studied interactions were recalculated using MMPBSA approach, which is relatively more reliable and accurate method in comparison to the MMGBSA approach, based on the trajectories obtained from performed molecular dynamics simulations. Based on the “delta total” values (Fig 6), which is final estimated binding free energy calculated from other presented energetic terms, compounds ZINC40467580 (-35.83 kcal/mol) and ZINC11692074 (-35.29 kcal/mol) had lower binding energies than reference ligand talazoparib (-30.55), while other two compounds ZINC170624334 (-26.72 kcal/mol) and ZINC6547121 (-25.28 kcal/mol) demonstrated higher binding energies. Remarkably all studied compounds in comparison to talazoparib demonstrated relatively lower values of electrostatic energy and van der Waals forces (Fig 6). However due to much higher electrostatic contribution of the studied compounds to the solvation free energy calculated by PB, overall “delta total” energy values of studied compounds are close to talazoparib. Differences in contribution of studied energetic terms to interaction of studied compounds, highlights additional interest and potential of selected compounds from drug design point of view.

## Conclusion

As the result of molecular docking and MMGBSA re-scoring experiments several chemical compounds with close or higher binding energies to the PARPi active site have been identified. The 2D chemical similarity analysis showed that the identified compounds include alternative to talazoparib chemical components and scaffolds. Differences in the interaction patterns of these compounds and talazoparib with amino acid residues of the active site of the catalytic domain of PARP1 enzyme indicate that these compounds might potentially have different therapeutically valuable properties. These compounds are of great interest for their further research as potential inhibitors of the PARP1 enzyme.

## Supporting information

S1 Table List of compounds with higher docking scores than talazoparib. (XLS)
